# Modification of Clinoptilolite as a Robust Adsorbent for Highly-Efficient Removal of Thorium (IV) from Aqueous Solutions

**DOI:** 10.3390/ijerph192113774

**Published:** 2022-10-23

**Authors:** Abdulrahman Masoud Alotaibi, Aznan Fazli Ismail

**Affiliations:** 1 Nuclear Science Programme, Faculty of Science and Technology, Universiti Kebangsaan Malaysia, UKM, Bangi 43600, Selangor, Malaysia; 2 Department of Physics, Faculty of Applied Science, Umm Al-Qura University, Makkah 21955, Saudi Arabia; 3 Nuclear Technology Research Centre, Faculty of Science and Technology, Universiti Kebangsaan Malaysia, UKM, Bangi 43600, Selangor, Malaysia

**Keywords:** adsorption, thorium, zeolite, clinoptilolite, Langmuir isotherm

## Abstract

The natural zeolite has been modified with sulphate and phosphate. The adsorption of thorium from the aqueous solutions by using the natural and modified zeolites has been investigated via a batch method. The adsorbent samples were characterized by X-ray Diffraction (XRD), N_2_ adsorption–desorption (BET), Fourier transform infrared (FTIR), field emission scanning electron microscopy (FESEM), and energy dispersive X-ray spectroscopy (EDX). Modification of natural zeolite with sulphate and phosphate was found to increase its adsorption capacity of thorium but reduced its specific surface area (S_BET_). The adsorption experiments were expressed by Langmuir, Freundlich and Dubinin–Radushkevitch (D–R) isotherm models and the results of adsorption demonstrated that the adsorption of thorium onto the natural and modified zeolites correlated better with the Langmuir isotherm model than with the Freundlich isotherm model. The maximum adsorption capacity (Qo) was determined using the Langmuir isotherm model at 25 °C and was found to be 17.27, 13.83, and 10.21 mg/g for phosphate-modified zeolite, sulfate-modified zeolite, and natural zeolite, respectively. The findings of this study indicate that phosphate-modified zeolite can be utilized as an effective and low-cost adsorbent material for the removal of thorium from aqueous solutions.

## 1. Introduction

Recently, there has been renewed interest in thorium’s possible application in the nuclear fuel cycle [1,2] as it has several advantages compared with uranium. Thorium is four times more abundant in nature than uranium and, therefore, potentially meets the substantial and expanding demand for energy resources across the world. Moreover. thorium is a naturally occurring radioactive heavy element, widely distributed in nature as an easily exploitable resource in many countries. It found in a variety of minerals, such as monazite, xenotime, zircon, and ilmenite [3,4,5]. Some human activities, such as nuclear fuel plants, ore mining, tin processing, rare-earth extraction process, production of phosphate fertilizer, phosphate rock processing, industrial boilers, coal-fired utilities, and laboratories dealing with radioactive substances, contributed to an increase in the concentration of thorium in our environment as a result of waste generated from such activities [6,7,8,9]. Thus, the removal of thorium from radioactive waste is regarded as a crucial issue in the treatment of such waste because it is extremely dangerous and can harm the environment and human health due to its radiotoxicity and chemical toxicity, as well as its long half-life [10]. In addition to this, the removal of thorium from radioactive waste by using an appropriate treatment will minimize the amount of waste that has to be disposed of, leading to a reduction in disposal costs and an improvement in the disposal site efficiency [11,12,13,14,15].

Treatment techniques used to remove radionuclides from radioactive wastes include adsorption, chemical precipitation, ion exchange, evaporation, reverse osmosis, micro-filtration, ultra-filtration, electrosorption, and others [10,16]. However, among these treatment techniques, adsorption is the most common technique because it is convenient, efficient, simple, inexpensive, has a large capacity, and produces no sludge. It has been extensively used to treat radioactive wastes [10,17]. Many types of materials have been utilized for thorium adsorption, such as activated carbon [18], gibbsite [19], illite [20], perlite [21], bentonite [22], and zeolite [23].

In the 1950s, zeolite was first considered for the treatment of radioactive wastes [24]. Zeolites have been employed in low level radioactive waste treatment techniques. However, zeolites have recently been used in the treatment of high and medium level wastes [25]. These materials have a high radiological stability to beta, alpha, and gamma irradiations, have a high ion exchange capacity, have an excellent chemical and thermal stability, with almost no reactivity to chemicals, are selective, affordable, and abundant. Moreover, the uniform presence of channels and pores in zeolite is an important advantage. That is why this mineral is known for its high absorption abilities, owing to its high surface-to-volume ratio. These unique properties make zeolites presently the most widely utilized adsorbents for the treatment of radioactive wastes [25,26,27]. Zeolites are crystalline aluminosilicates with three-dimensional crystal frameworks built of tetrahedral silica (SiO_4_) and alumina (AlO_4_) which are connected to each other via sharing oxygen atoms. There are many types of natural zeolites known around the world, such as mordenite, clinoptilolite, chabazite, phillipsite, analcime, laumontite, and stilbite [28].

Among the 40 identified types of natural zeolites, clinoptilolite is considered the most important and abundant natural zeolite. It is found in large deposits all around the world and is widely utilized on a global scale in different sorption treatment studies of industrial wastewater [29,30]. Clinoptilolite is a high silica member of the heulandite group natural zeolites. The typical chemical formula for clinoptilolite is given by Na_6_[(AlO_2_)_6_(SiO_2_)_30_]·24 H_2_O [31]. However, this chemical composition of clinoptilolite is generally variable in both the extra framework cation number and its framework [32]. The types and physical and chemical properties of natural clinoptilolites rely on the environment and the place of the deposits [33]. The low cost, availability, and favorable ion exchange capacity of clinoptilolite which make it particularly suitable to be used as an adsorbent material for environmental protection. The cost of the clinoptilolite would be negligible in comparison with the cost of activated carbon, as the cost of clinoptilolite is approximately 0.06–0.08 US $ per kg [32,34,35,36].

There are many researchers and scientists looking for ways to enhance the adsorption of thorium through modifying the surface of natural zeolite. From the point of view of adsorption performance and cation exchange capacity, modified zeolites have higher performance and capacity than the natural zeolites [36,37,38]. Several studies on zeolite modification have been conducted in order to further improve its adsorption efficiency for thorium removal [39,40,41,42]. However, to the best of our knowledge, there are no reports on the removal of thorium from an aqueous solution using natural zeolite modified with sulphate and phosphate. The adsorption of phosphate and sulphate anions would increase the negative charge on the natural zeolite surface and, as result of that, the removal efficiency of zeolite towards thorium could be increased.

In the present study, the natural zeolite (clinoptilolite) has been modified with sulphate and phosphate in order to enhance the removal of thorium ions from aqueous solutions. Here, X-ray Diffraction (XRD), N_2_ adsorption–desorption (BET), Fourier transform infrared (FTIR), field emission scanning electron microscopy (FESEM), and EDX were used to characterize the natural zeolite (NZ), phosphate-modified zeolite (PZ), and sulfate-modified zeolite (SZ). The equilibrium adsorption data have been analyzed using common isotherm models, namely Langmuir, Freundlich, and Dubinin–Radushkevitch (D–R), and the equilibriums parameters have been calculated. The findings acquired in this investigation may be useful for future research and demonstrate the practical uses of the adsorbent in the remediation of thorium industrial residue.

## 2. Materials and Methods

### 2.1. Materials

The natural zeolite (clinoptilolite) used in this research was supplied by Heiltropfen (Heiltropfen Lab. LPP, 27 Old Gloucester Street, WC1N 3AX, London, UK). According to the manufacturer, the product is 100% natural volcanic mineral from Slovakia, EU, with more than 90% clinoptilolite content. Potassium dihydrogen phosphate (KH_2_PO_4_, ≥99.0%, M_w_ = 136.09 g/mol) and sodium sulfate (Na_2_SO_4_, anhydrous, ≥99.0%, M_w_ = 142.04 g/mol) that were used for surface modification of the natural zeolite were purchased from Sigma-Aldrich. The stock standard solution of 1000 mg/L of thorium nitrate [Th(NO_3_)_4_] was purchased from AccuStandard, New Haven-CT, USA. Different concentrations of thorium solution were prepared from the stock solution by appropriate dilution. Barium chloride dihydrate (BaCl_2_, ≥99.0%, M_w_ = 244.26 g/mol) and iron (III) chloride (FeCl_3_, anhydrous, ≥99.0%, M_w_ = 162.20 g/mol), which were used to test for the ions of sulphate and phosphate, were purchased from Sigma-Aldrich. Dilute solutions of sodium hydroxide (NaOH) and nitric acid (HNO_3_) were used to adjust the pH to the required value. All materials and chemicals were used as obtained from the suppliers without additional purification and modification, unless stated.

### 2.2. Modification and Characterization of the Adsorbent Materials

**Figure 1:**
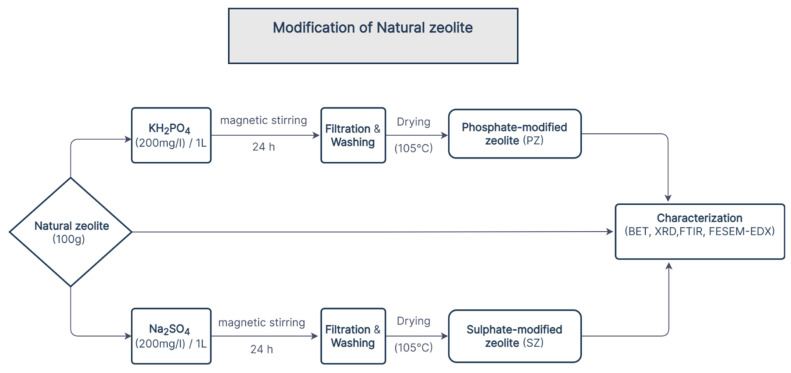
Simplified flowchart illustrating the modification procedure of natural zeolite using sulphate and phosphate.

The modification of the natural zeolite (clinoptilolite) was carried out by mixing 100 g of the natural zeolite (clinoptilolite) with 1000 mL of 200 mg/L of sodium sulfate (Na_2_SO_4_) and potassium dihydrogen phosphate (KH_2_PO_4_) in a 2000 mL beaker. The zeolite suspensions were stirred on a magnetic stirrer (a Cimarec 1 model, Thermolyne Barnstead, Dubuque, IA, USA) for 24 h at room temperature (25 °C), after which they were been filtered off by using Whatman filter papers (Whatman’s No. 1, GE Healthcare Life Science, Hatfield, UK). The solid phase was washed several times with 1000 mL portion of deionized water in order to remove excess sulphate and phosphate ions. Tests for sulphate and phosphate in the filtered solution were confirmed negative. The samples of PZ and SZ were subsequently dried in an oven at 105 °C and were packed into glass containers and stored in the desiccator at room temperature for further use. The modification procedure of natural zeolite using sulphate and phosphate is illustrated in Figure 1.

The natural and modified adsorbents were characterized using X-ray diffraction (XRD, Bruker AXS Germany, a D8 Advance model, fitted with a scintillation counter) to confirm the identity, purity, and crystallinity of the adsorbent materials. The X-ray diffraction (XRD) data of the natural and modified zeolites were collected using a Bruker AXS Advance D8 diffractometer with a monochromatic Cu Kα radiation source (wavelength (λ) = 1.5406 Å) consisting of Soller slits (0.02 rad), a fixed divergence slit (0.3°), and operated at a tube current of 40 mA and a tube voltage of 40 kV with a scanning speed of 2°/min, at room temperature. The samples were scanned between 2θ = 5° and 50° with a scintillation counter detector.

The specific surface area (S_BET_) of the adsorbent materials has been determined by using a micromeritics accelerated surface area and porosimetry analyzer system (ASAP 2020, Micromeritics, Atlanta, GA, USA), while the nitrogen adsorption/desorption isotherms were measured at 77 K. The natural and modified adsorbents were first degassed under vacuum conditions at 300 °C to avoid damaging the structure of zeolite for 10 h before conducting the BET analysis. The nitrogen adsorption/desorption isotherm measurements were recorded at relative pressures (P/Po^−1^) between 0.01 and 0.99. The specific surface area (S_BET_) of the adsorbent materials was determined using the BET method. The pore size distributions were determined from the desorption branch using the Barrett–Joyner–Halenda (BJH) method.

Fourier transform infrared spectroscopy of the natural and modified adsorbents has been studied using an FTIR analysis instrument (Perkin Elmer, spectrum 400 FT-IR NIR spectrometer) to identify the functional groups present in the adsorbent materials. This instrument is equipped with an ATR single reflection diamond crystal. The Fourier transform infrared spectra (FTIR) of the natural and modified zeolite samples were collected in the range of 650–4000 cm^−1^.

The surface morphology of the adsorbent materials has been observed by using field emission scanning electron microscopy (FESEM, Merlin ZEISS GEMINI 2, Oberkochen, Germany) under the following analytical conditions: EHT = 3.00 kV, Signal A = SE2, WD = 8.9, 9.2, and 9.3 mm at different magnifications (1000×, 10,000×, 20,000×, and 30,000×), respectively. The field emission scanning electron microscopy coupled with energy dispersive X-ray spectroscopy (EDX) was used to ensure the modification procedure of the natural zeolite and to determine the dispersion of desirable species in the adsorbent materials. Concentrations and positions of different elements have been determined using X-ray elemental mapping (dot mapping) analysis.

### 2.3. Adsorption Experiments

**Figure 2:**
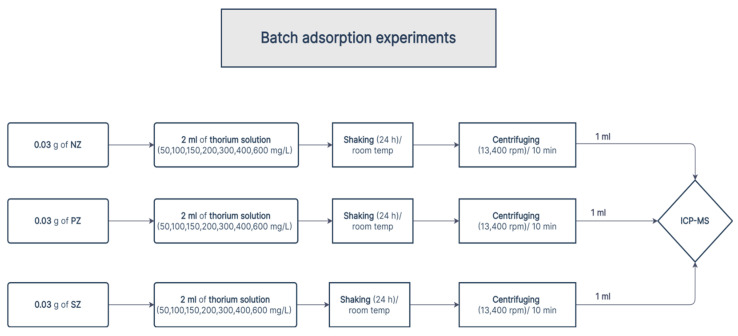
The schematic illustration of the batch adsorption experiments.

The adsorption experiments were carried out using the batch method. In these batch adsorption experiments, accurately weighed masses (~0.03 g) of natural zeolite (clinoptilolite), PZ, and SZ were placed in 2 mL polypropylene vials, and an aliquot (2 mL) of each different concentration (50, 100, 150, 200, 300, 400, and 600 mg /L) of thorium solution was added. The polypropylene vials were directly placed vertically on a shaker (high-speed microplate shaker, Illumina, a 945190 model, San Diego, CA, USA) at 775 rpm for one day to reach equilibrium at room temperature (25 °C), at a constant pH (3), being controlled by using a diluted solution of sodium hydroxide NaOH (1M) or nitric acid HNO3 (1M). A pH meter (a EUTECH ph700 model, Thermo Scientific, Waltham, WA, USA) was used to monitor the pH value. The aqueous phase was separated by using centrifugation (with a model TGL-16 C centrifuge, Shanghai Longyue Instrument Equipment Co., Ltd., Shanghai, China) at 13,400 rpm for ten minutes. One milliliter of the supernatant was taken from each polypropylene vial to be analyzed using inductive coupled plasma–mass spectrometry (ICP–MS, Perkin Elmer Sciex Elan 9000, USA) in order to determine the equilibrium concentration of thorium remaining in the solutions after the adsorption processes. The schematic illustration of the batch adsorption experiments is presented in Figure 2.

All experiments were conducted in triplicate, and only the average results were reported. The difference between the initial and equilibrium concentration of thorium has been used to calculate the amount of the thorium adsorbed on the surface of the adsorbent (qe) by using the following Equation (1):

(1) $$\mathrm{qe}=\frac{\left(\mathrm{Co}-\mathrm{Ce}\right)\;V\;}{m}$$

where Co is the initial concentration of the adsorbate molecules in the solution (mg/L), Ce is the equilibrium concentration of adsorbate molecules remaining in the solution (mg/L), V is the volume of solution (L), m is the mass of absorbent material (g), and qe is the adsorption capacity of adsorbent (mg/g).

The removal efficiency of the adsorbent material can be obtained using the following Equation (2) [10]:

(2) $$\mathrm{The}\;\mathrm{removal}\;\mathrm{efficiency}\;\left(\%\right)=\frac{\left(\mathrm{Co}-\mathrm{Ce}\right)}{\mathrm{Co}}\times 100$$

## 3. Results and Discussion

### 3.1. Characterization of Adsorbents

#### 3.1.1. X-ray Diffraction (XRD) Analysis of Adsorbents

**Figure 3:**
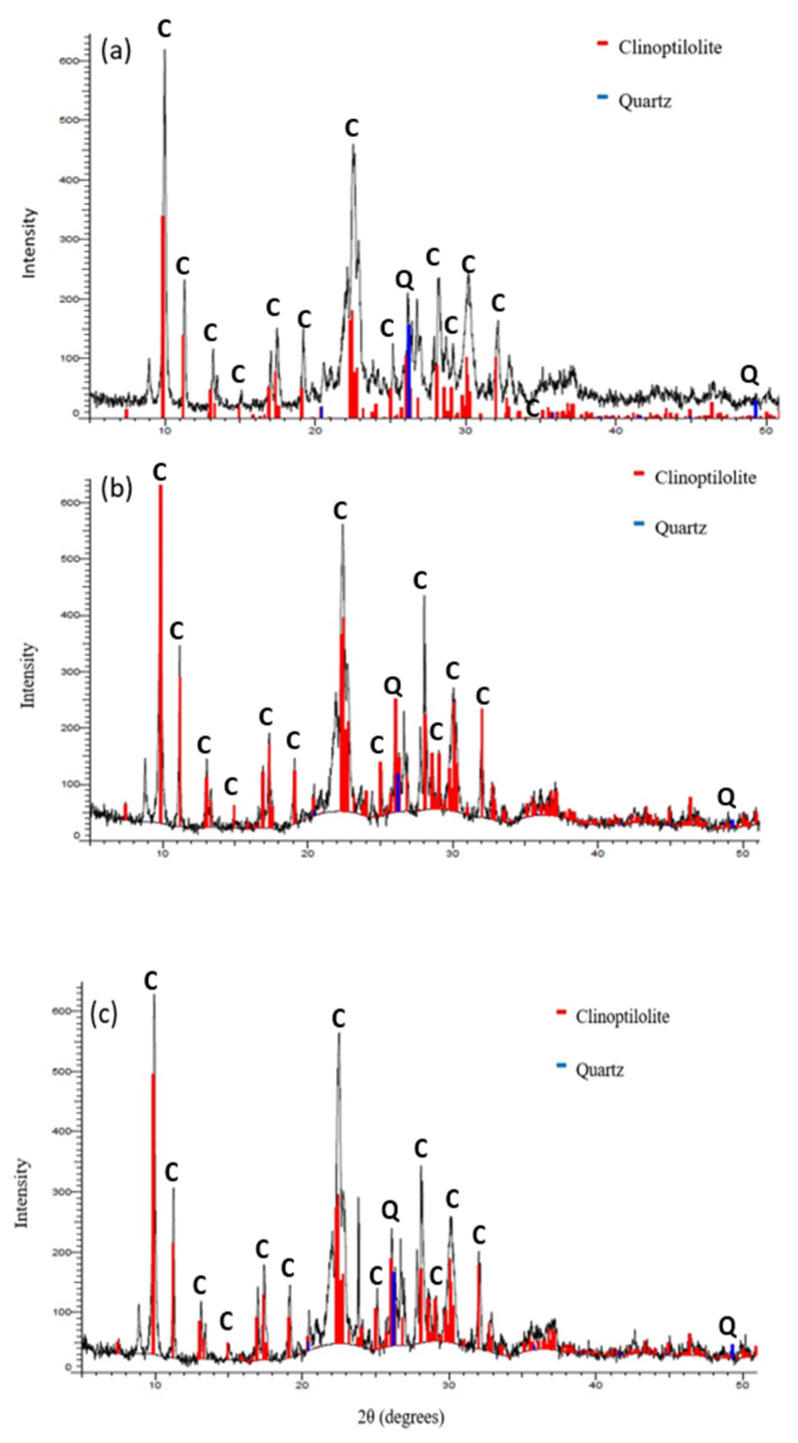
The X-ray diffraction (XRD) patterns of (**a**) NZ, (**b**) PZ, and (**c**) SZ.

The X-ray diffraction (XRD) patterns of NZ, PZ, and SZ are shown in Figure 3. The results of the X-ray diffraction (XRD) patterns have been compared with a database of diffraction patterns supplied by the International Center for Diffraction database (ICDD) using Diffrac.Suite Eva software. It can be seen that all samples contained clinoptilolite as the major mineral, which is defined by its principal characteristic peaks at 2θ = 9.87, 11.18, 13.05, 13.33, 14.93, 16.90, 17.34, 19.08, 22.48, 28.12, 30.03, and 31.99° according to (01-083-1261 JCPDS card) [43]. The XRD patterns of NZ, PZ, and SZ also showed that there is a small amount of a quartz (01-075-1555 JCPDS card) phase as an impurity [44].

**Figure 4:**
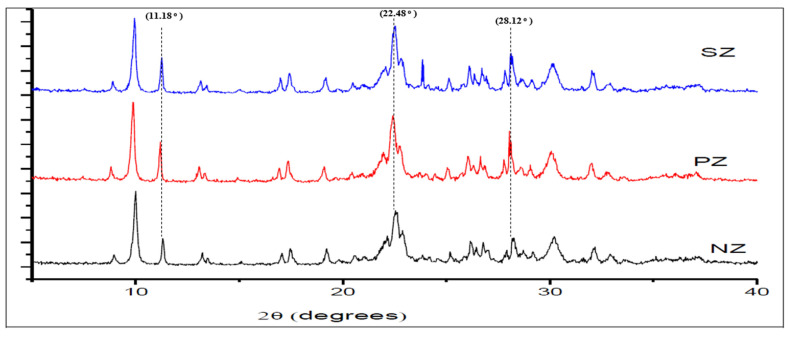
The close-up X-ray diffraction (XRD) patterns of NZ, PZ, and SZ after several washings with deionized water.

Moreover, comparative analysis of the XRD patterns did not show considerable changes in the positions of most of the peaks after the modification using sulphate and phosphate. Hence, the results showed that the modification using sulphate and phosphate had no effect on the primary structure of natural zeolite. However, an increase can be observed in the intensity of the peaks for PZ and SZ at 2θ = 11.18°, 22.48° and 28.12° as shown in Figure 4. Given that the natural zeolite is a mineral that was mined from deposits, the presence of dust and minor impurities in the zeolite phase is common. As a result of many repeated washings with deionized water during the process of modification, these impurities have been removed from the structure of natural zeolite and, as a result, the intensity of the peaks and crystallinity of natural zeolite are increased. The crystallinity of NZ, PZ, and SZ was 82.36%, 86.38%, and 85.02%, respectively. A similar increase in the intensity of the peaks of nature zeolite was also observed by other researchers [44].

#### 3.1.2. N_2_ Adsorption–Desorption Isotherm Analysis

In Table 1, the textural properties, such as the specific surface area (S_BET_), average pore size, and total pore volume of NZ, PZ, and SZ are reported. As shown in Table 1, the specific surface area (S_BET_) of the natural zeolite (NZ) is 28.84 ± 0.49 m^2^/g. The low specific surface area (S_BET_) indicates that the molecules of nitrogen have difficulty accessing the micropore volume of NZ at 77 K [45]. This value is comparable with other published studies on the natural zeolite (clinoptilolite) samples from different origins, such as 29.91 m^2^/g for a sample from the Nižný Hrabovec Mine (Slovakia) [46], ~28 m^2^/g from Petrota village (Northern Thrace, Greece) [45] and 20.03 m^2^/g from the Caimanes deposit (Cuba) [47].

However, after surface modification of the natural zeolite, the specific surface area (S_BET_) of the natural zeolite was reduced by 13.2% in the case of modification using phosphate and by 28.5% in the case of modification using sulphate as presented in Table 1. This reduction in the specific surface area (S_BET_) has previously been observed and reported by many other researchers as well [48,49,50]. The modification with sulphate and phosphate may have blocked some of the pores in the natural zeolite, which eventually led to this reduction in the specific surface area (S_BET_).

**Figure 5:**
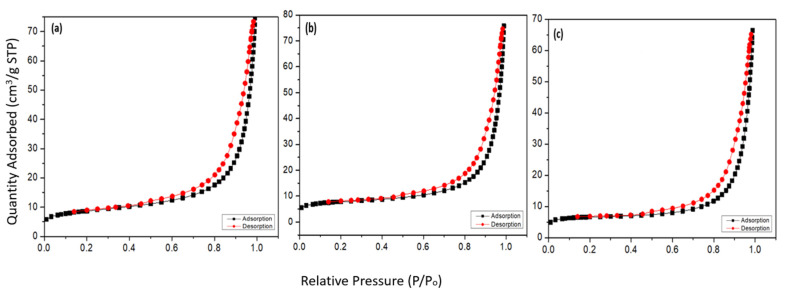
The nitrogen adsorption/desorption isotherms of (**a**) NZ, (**b**) PZ, and (**c**) SZ.

In Figure 5, the nitrogen adsorption/desorption isotherms of NZ, PZ, and SZ are visualized. According to the International Union of Pure and Applied Chemistry (IUPAC) for porous materials, these adsorption/desorption isotherms obtained can be classified as adsorption/desorption isotherm type IV, which is the characteristic of mesoporous materials. All of these nitrogen adsorption/desorption isotherms of NZ, PZ, and SZ have a type H3 hysteresis loop, in the range 0.4–0.99 P/Po. The existence of this type of hysteresis loop is considered to be a characteristic for the clinoptilolite material, which can be attributed to multilayer physical adsorption followed by capillary condensation either in the space between the crystallites of zeolite or in mesopores of impurities (quartz, feldspars, etc.) [45,51,52].

**Figure 6:**
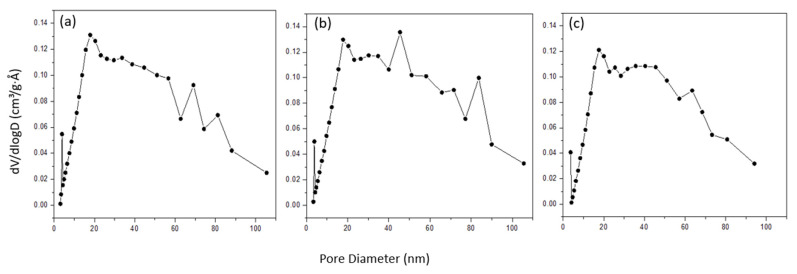
The pore size distribution of (**a**) NZ, (**b**) PZ, and (**c**) SZ.

Figure 6 shows the pore size distribution of NZ, PZ, and SZ, as derived from the desorption branch of the nitrogen adsorption/desorption isotherms based on the BJH method. According to the IUPAC classification, pores can be divided into three main categories, namely micropores (diameter < 2 nm), mesopores (2–50 nm), and macropores (diameter >50 nm). As seen in Figure 6, the analysis of pore size suggests that NZ, PZ, and SZ are mesoporous materials, as most of their pore diameters are in the range of 2 to 50 nm.

#### 3.1.3. Fourier Transforms Infrared (FTIR) Analysis of Adsorbents

**Figure 7:**
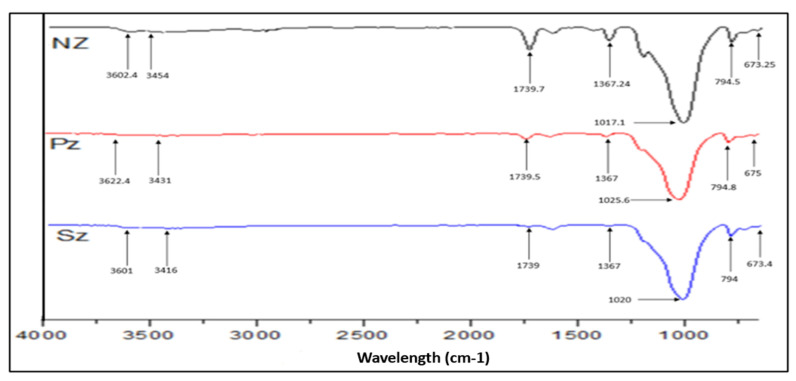
The FTIR spectrum of NZ, PZ, and SZ.

The FTIR spectrum of NZ, PZ, and SZ have been shown in Figure 7. Based on the spectrum of the natural zeolite, the functional groups that exist are the –OH group in the wavenumber 3454 cm^−1^, which corresponded to stretching vibration; the H-O-H in the wavenumber 1628.5 cm^−1^, which corresponded to harmonic vibration; the (Si, Al)O_4_ in 1203.43 cm^−1^; and the T-O (T=Si or Al) in wavenumber 1017.1 cm^−1^. These functional groups are summarized in Table 2. The peaks identified on the natural zeolite spectra are similar to the results of infrared testing of some zeolites [53,54].

Modification of natural zeolite resulted in a shift in the structural –OH vibrational band from 3602.4 and 3454 cm^−1^ to 3622.4 and 3431 cm^−1^, respectively, for phosphate-modified zeolite and to 3601 and 3416 cm^−1^, respectively, for sulphate-modified zeolite. The stretching and bending vibrations in natural zeolite shifted from 1017.1 cm^−1^ to 1025.6 cm^−1^ for phosphate-modified zeolite and 1020 cm^−1^ for sulphate-modified zeolite. The 1025.6 and 1020 cm^−1^ are the P–O and S–O stretching peaks, respectively. This stretching vibration of the P–O group is usually observed between 1000 and 1120 cm^−1^ [55], while the asymmetric S–O mode occurs at around 1080 cm^−1^ and the symmetric S–O is found at around 1020 cm^−1^ [56]. Usually both peaks appear as a strong band, as seen in Figure 7. For a good comparison, the locations of the corresponding bands were shown in Table 3. According to the analysis of the earlier FTIR spectra, it is, therefore, very possible to conclude that the modification of natural zeolite with sulphate and phosphate was effective on the surface of the natural zeolite. 

#### 3.1.4. Field Emission Scanning Electron Microscopy (FESEM) and EDX Analysis

**Figure 8:**
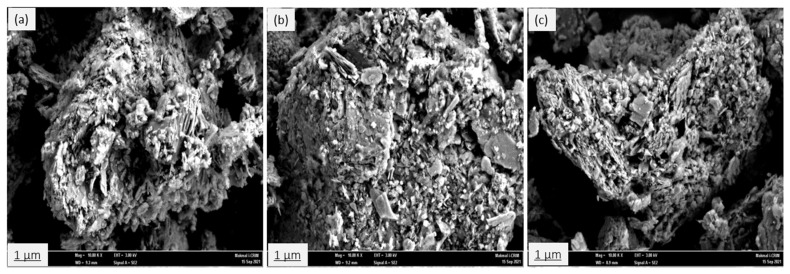
FESEM micrographs of (**a**) NZ, (**b**), PZ, and (**c**) SZ, at a magnification of 10,000×.

The surface morphology of the natural zeolite (clinoptilolite) sample before and after modification has been investigated using field emission scanning electron microscopy (FESEM) equipped with energy dispersive X-ray spectroscopy (EDX). Figure 8a–c represent the surface morphology of NZ, PZ, and SZ. The FESEM images clearly show that the crystals of the samples appear as plates and laths. This observation is in agreement with those obtained by [57]. The micrographs reveal that there are no substantial changes in the surface morphology of NZ after modification, as the particle and pore sizes of PZ and SZ are quite similar compared to NZ.

**Figure 9:**
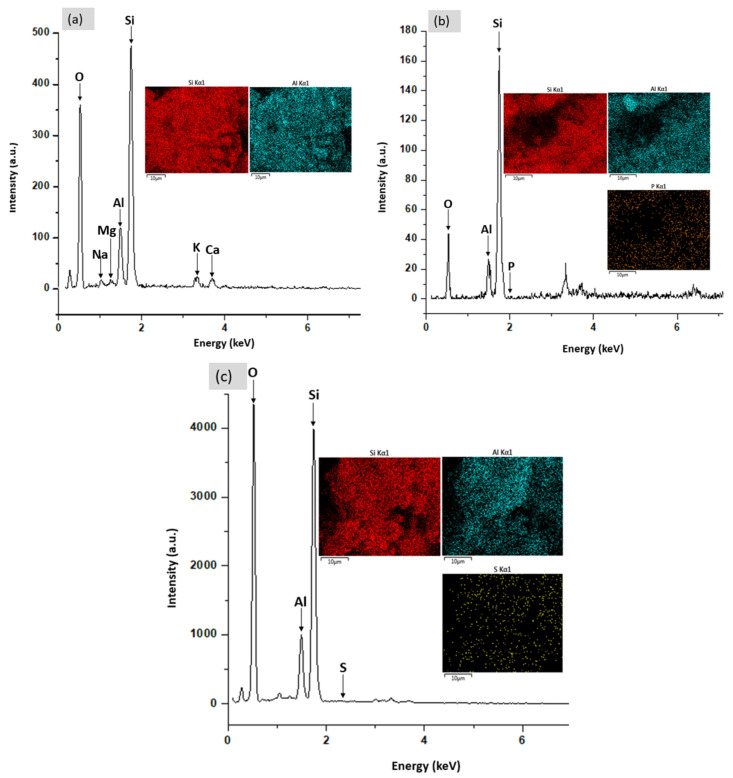
EDX analysis showing the EDX spectrum and dot mapping of (**a**) NZ, (**b**) PZ, and (**c**) SZ.

The EDX spectrum and dot mapping of NZ, PZ and SZ are presented in Figure 9 while the elemental composition is given in Table 4, respectively. The results of the EDX analysis show that the dominant exchangeable cations in the structure of the natural zeolite (NZ) were found to be Na^+^, K^+^, Mg^2+^, and Ca^2+^ as shown in Figure 9a. The results clearly confirm the presence of the P element in the structure of phosphate-modified zeolite and the S element in the sulphate-modified zeolite structure. These results are consistent with results obtained from FTIR analysis. Thus, according to the analysis of EDX and the element mapping, it can be proved that the modification of the natural zeolite with sulphate and phosphate was successful on the zeolite surface. Regarding elemental analysis, the elemental composition of NZ, PZ, and SZ has been recorded by EDX.

### 3.2. Adsorption Isotherms

An adsorption isotherm is a very useful curve that is most commonly utilized for describing the adsorption process, and which expresses the relationship between the amount of the adsorbate molecules adsorbed on the surface of the adsorbent and the equilibrium concentration of adsorbate molecules remaining in the solution at a constant temperature [17]. Several adsorption isotherm models have been proposed and applied in the study of the adsorption process. Among these isotherm models, the Langmuir and Freundlich isotherm models are the most widely utilized ones [58]. In this report, the Langmuir, Freundlich, and Dubinin–Radushkevitch (D–R) models were used to analyze adsorption equilibrium data.

#### 3.2.1. Langmuir Isotherm

**Figure 10:**
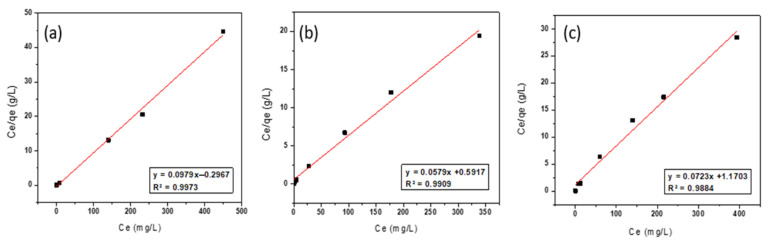
Langmuir isotherm model of thorium adsorption onto (**a**) NZ, (**b**) PZ, and (**c**) SZ.

The Langmuir adsorption isotherm model is based upon the assumption that the adsorption process occurs on a homogeneous surface with all adsorption sites on the surface being equivalent. Furthermore, only one monolayer of adsorbate molecules is formed when the adsorption reaches its maximum, with no interaction between them on the surface of the adsorbent. The linear form of the Langmuir adsorption isotherm model can be described by the following Equation (3) [59]:

(3) $$\frac{\mathrm{Ce}}{\mathrm{qe}}=\frac{1}{\mathrm{Qo}\;\times K_{L}}+\left[\frac{1}{\mathrm{Qo}\;}\right]\mathrm{Ce}$$

where Ce is the equilibrium concentration of adsorbate molecules in the solution (mg/L), qe is the adsorption capacity of adsorbent (mg/g), Qo is the maximum adsorption capacity for forming one layer (mg/g), and K_L_ is the Langmuir isotherm coefficient (L/mg). The plot of Ce/qe against Ce gives a linear relationship of which the slope is equal to (1/Qo), and intercept (1/Qo × K_L_). In Figure 10, the values of Ce/qe were plotted against the equilibrium concentrations of thorium Ce. Langmuir isotherm model parameters and the correlation coefficient values (R^2^) were calculated and presented in Table 5.

**Figure 11:**
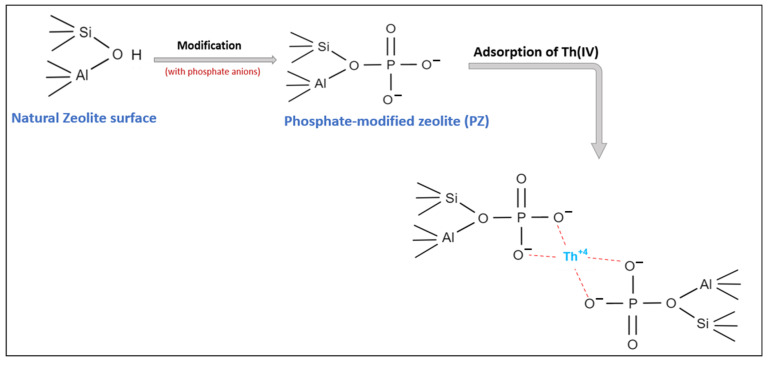
Schematic diagram of phosphate interaction with natural zeolite and the proposed mechanism of thorium adsorption onto phosphate-modified zeolite (PZ).

As shown in Table 5, the Langmuir isotherm model is a better fit for describing the adsorption of thorium onto NZ, PZ, and SZ than the Freundlich and Dubinin–Radushkevitch (D–R) isotherm models (R^2^ = 0.9973, R^2^ =0.9909 and R^2^ = 0.9884, respectively). The maximum adsorption capacity (Qo) of NZ, PZ, and SZ for thorium are found to be 10.21, 17.27 and 13.83 mg/g, respectively, which are in a good agreement with the experimental data. Although the modification of natural zeolite with sulphate and phosphate decreased the specific surface area (S_BET_), the adsorption capacity of natural zeolite was enhanced. This is due to the fact that the modification of natural zeolite by using sulphate and phosphate anions increases the negative charge on the surface of natural zeolite by introducing additional adsorption sites, thereby increasing its maximum adsorption capacity for thorium. This result is in line with what other researchers have reported [49,50,60,61,62]. The presented results show that phosphate-modified zeolite (PZ) displayed a higher maximum adsorption capacity than sulphate-modified zeolite (SZ), which is concordant with the results obtained by [60,63,64]. This may be attributed to the fact that modification with phosphate anions would provide more negative charges (i.e., two negative charges per phosphate anion) on the surface of natural zeolite for thorium adsorption compared to modification with sulphate anions. The mechanism of interaction of phosphate anions added to the natural zeolite, and the proposed mechanism of thorium onto phosphate-modified zeolite (PZ) in this research, is schematically illustrated in Figure 11. Phosphate anions might bind to bridging oxygen groups on the framework of natural zeolite during the interaction of phosphate anion with natural zeolite [65,66,67,68,69,70], and each phosphate anion would offer two negative charges on the surface of natural zeolite. Thus, two phosphate anions could be able to form a complex with the thorium ion, as shown in Figure 11.

**Figure 12:**
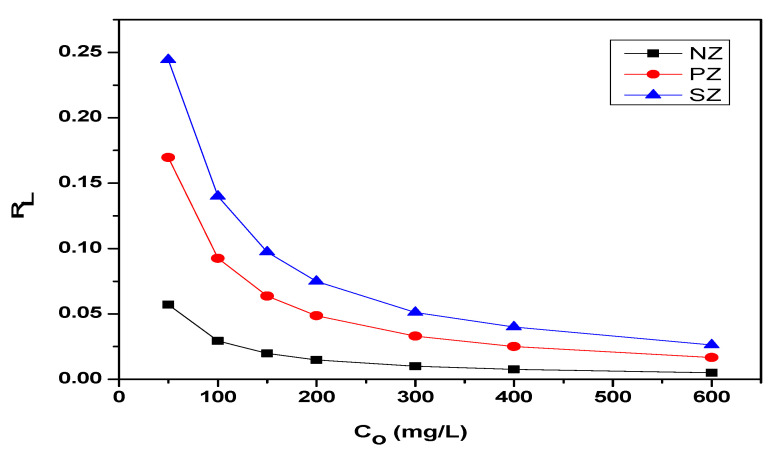
The separation factor (R_L_) for the adsorption of thorium onto NZ, PZ, and SZ.

The important features of the Langmuir adsorption isotherm model can be expressed in terms of the parameter of dimensionless equilibrium, usually known as the separation factor (R_L_) which is defined by Webber and Chakkravorti and can be represented by the following Equation (4) [71]:

(4) $$R_{L}=\frac{1}{\left(1+K_{L}\times \mathrm{Co}\right)}$$

where Co is the initial concentration of the adsorbate molecules in the solution (mg/L) and K_L_ is the Langmuir isotherm coefficient (L/mg). The values of the dimensionless equilibrium parameter (R_L_) indicate the nature of the adsorption to be either unfavorable at (R_L_ > 1), favorable at (0 < R_L_ < 1), irreversible at (R_L_ = 0), or linear at (R_L_ = 1) [72]. The calculated R_L_ values for all of the adsorbents in this study are listed in Table 5. A comparison of the RL values for the adsorption of thorium onto NZ, PZ, and SZ at the initial concentrations of thorium in the range of 50–600 mg/L is displayed in Figure 12. The calculated data reveal that all the R_L_ values for the adsorption of thorium onto the studied adsorbents are less than one and higher than zero (0 < R_L_ < 1), which indicates that these adsorbents are favorable for the adsorption of thorium under the selected conditions.

#### 3.2.2. Freundlich isotherm

**Figure 13:**
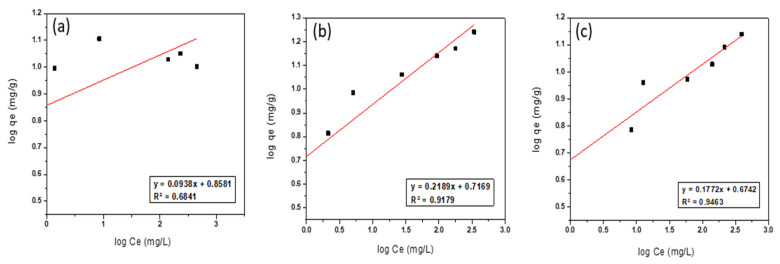
Freundlich isotherm model of thorium adsorption onto (**a**) NZ, (**b**) PZ, and (**c**) SZ.

On the contrary, the Freundlich adsorption isotherm model assumes that the process of adsorption is multilayer adsorption, which takes place on heterogeneous surfaces and involves interactions between the adsorbate molecules on the adsorbent surface. The linear form of the Freundlich adsorption isotherm model can be represented by the following Equation (5) [73]:

(5) $$\log\;\mathrm{qe}\;={\log\;K}_{f}+\left(1/n\right)\;\log\;\mathrm{Ce}$$

where qe is the adsorption capacity of the adsorbent (mg/g), K_f_ is the Freundlich constant (mg/g), which is indicative of the capacity of adsorption, n is the Freundlich constant (unitless), which is indicative of the intensity of the adsorption process, and Ce is the equilibrium concentration of adsorbate molecules in the solution (mg/L). The plot of log qe versus log Ce will yield a linear relationship of which the slope is equal to (1/n) and the intercept (log K_f_). In Figure 13, the values of log qe were plotted against log Ce. Freundlich parameters and the correlation coefficient (R^2^) values were calculated and presented in Table 5.

From Table 5, The correlation coefficient values (R^2^) for the Freundlich isotherm model (≤0.94) are lower compared with the R^2^ values for the Langmuir isotherm model (≥0.98). Moreover, the adsorption capacities (K_f_ value) of NZ, PZ, and SZ is 7.21, 5.21, and 4.72 mg/g, respectively, which are lower than the experimental data of these adsorbents for thorium adsorption at room temperature. Therefore, it can be said that the Freundlich isotherm model does not fit the adsorption of thorium onto NZ, PZ, and SZ.

#### 3.2.3. Dubinin–Radushkevich Isotherm (D–R)

**Figure 14:**
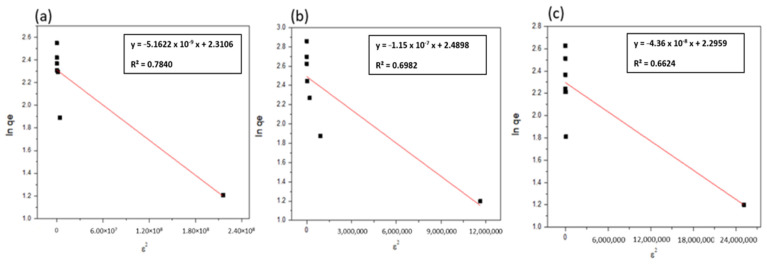
Dubinin–Radushkevitch (D–R) model of thorium adsorption onto (**a**) NZ, (**b**) PZ, and (**c**) SZ.

The Dubinin–Radushkevitch (D–R) isotherm model is more general than the Langmuir isotherm model since it does not postulate a constant adsorption potential or a homogeneous surface. The D–R isotherm model can be utilized to explain adsorption on both heterogeneous and homogenous surfaces [32,74]. The linearized form of the D–R isotherm model equation can be expressed by the following Equation (6) [74]:

(6) $$\mathrm{In}\;\mathrm{qe}={\mathrm{In}\;q}_{\max}-{K\;\varepsilon }^{2}$$

where qe is the adsorption capacity of adsorbent (mg/g), q_max_ is the maximum adsorption capacity (mg/g), K is a constant related to the energy of adsorption (mol^2^/kJ^2^), and ε is Polanyi potential (kJ/mol), which can be described as in the following Equation (7):

(7) $$\varepsilon =\;\mathrm{RT}\;\mathrm{In}\left(1+\frac{1}{\mathrm{Ce}}\right)$$

where R is the gas law constant and is equal to 8.314 (kJ/mol·K), T is the absolute temperature (K), and Ce is the equilibrium concentration of adsorbate molecules in the solution (mg/L). The plot of In qe against ε^2^ gives a linear relationship of which the slope is equal to K, and the intercept (In q_max_). In Figure 14, the values of In qe were plotted against ε^2^. The Dubinin–Radushkevitch (D–R) constants (qmax, K) and the correlation coefficient values (R^2^) were calculated and presented in Table 5.

Table 5 showed the qmax values from D–R isotherm for thorium ions, and the difference in the maximum adsorption capacity values derived from the D–R and Langmuir isotherm models can be attributed to the two models’ different definitions of the maximum adsorption capacity. In the D–R model, q_max_ denotes the maximum thorium ion adsorption at the adsorbent’s total specific microspore volume, whereas in the Langmuir model, Qo represents the maximum thorium ions adsorption at the monolayer coverage. Therefore, the maximum adsorption capacity values derived from the Langmuir isotherm model are higher than the values derived from the D–R model [3].

### 3.3. Removal Efficiency of Thorium Ions

**Figure 15:**
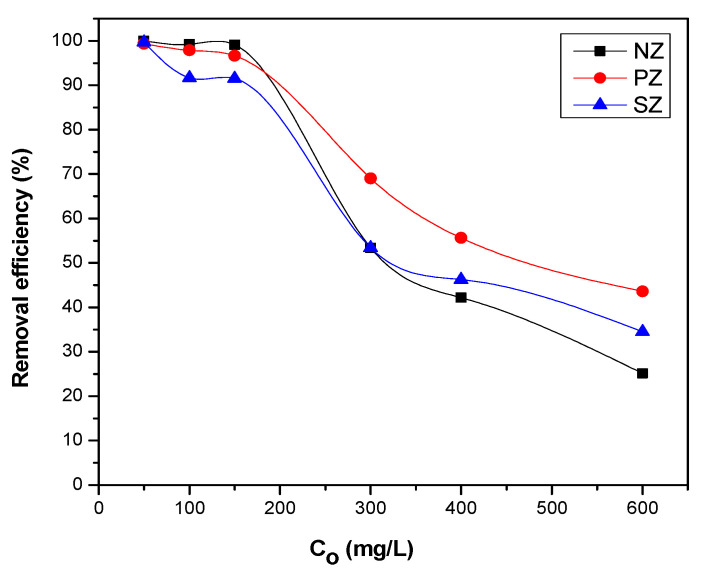
The thorium removal efficiency by NZ, PZ, and SZ using various initial thorium concentrations. Experimental conditions were as follows: adsorbent media 0.03 g/2 mL; pH 3 and 24 h as contact time at room temperature 25 °C.

The influence of the initial thorium concentration on the removal efficiency of thorium ions by NZ, PZ, and SZ is illustrated in Figure 15. The adsorption experiments were performed for 24 h at various thorium contents (50–600 mg/L) while the other parameters remained constant. The results revealed a decrease in the thorium removal efficiency by increasing the initial concentration of thorium. This could be attributed to the adsorption sites on the surface of adsorbents being saturated [75]. It can be observed that modified zeolites show higher removal efficiency for thorium than unmodified zeolite at high thorium concentrations. This enhancement in the removal efficiency may be due to the generation of additional adsorption sites by modifying natural zeolite [50].

### 3.4. Characterization of the Adsorbent Materials Post Adsorption Process

**Figure 16:**
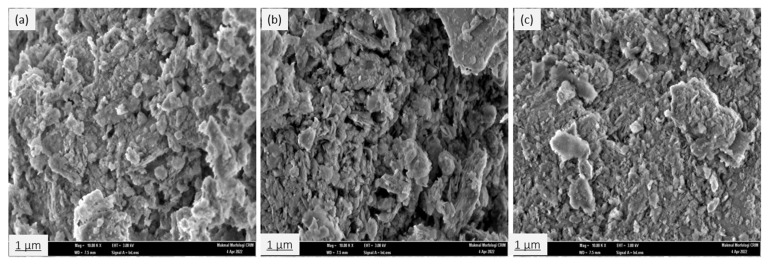
The FESEM image of (**a**) NZ, (**b**) PZ, and (**c**) SZ after the adsorption of thorium, at a magnification of 10,000×.

**Figure 17:**
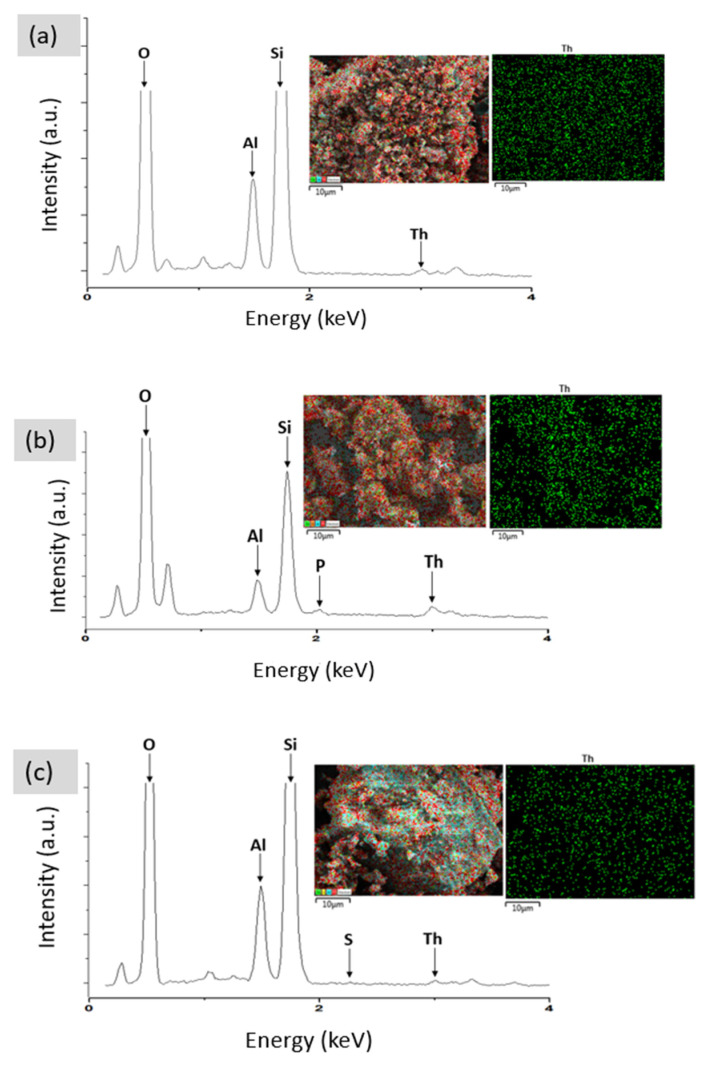
EDX analysis showing the EDX spectrum and dot mapping of (**a**) NZ, (**b**) PZ, and (**c**) SZ after the adsorption of thorium, at a magnification of 10,000×.

The adsorbent materials were subjected to further FESEM–EDX analyses to verify the existence of thorium ions on NZ, PZ, and SZ. The surface morphology of the adsorbents after the adsorption of thorium is shown in Figure 16. In these images, the bright areas might refer to the accumulation of thorium on the surface of the adsorbents [23]. The EDX spectrum and elemental mapping confirm the adsorption of thorium onto NZ, PZ, and SZ, as shown in Figure 17. The elemental mapping shows that the thorium ions are adsorbed in a random manner.

### 3.5. Comparison of Thorium Adsorption Capacity of PZ and SZ with other Adsorbents

Table 6 shows a comparison of the thorium adsorption capabilities of various adsorbents in the aqueous solutions under different experimental conditions. It can be clearly seen from this table that the maximum adsorption capacity of thorium onto PZ is better than that of the majority of other reported adsorbents. These findings indicate that phosphate-modified zeolite (PZ) has promising potential as an alternative adsorbent for thorium removal from the aqueous solutions.

## 4. Conclusions

In the light of this research, the following conclusions can be drawn:

- The results of the FTIR and EDX analysis provided strong evidence that the modification of the natural zeolite surface with sulphate and phosphate anions was successful and effective;
- Surface modification of natural zeolite with sulphate and phosphate anions increased its adsorption capacity and the adsorption of thorium from the aqueous solutions but decreased the specific surface area slightly. The thorium ions adsorption was more effective on PZ;
- The adsorption equilibrium data were analyzed using the Langmuir, Freundlich, and Dubinin–Radushkevitch (D–R) isotherm models, and adsorption data revealed that the adsorption of thorium onto the natural and modified zeolites was better fitted to the Langmuir isotherm model than the Freundlich or Dubinin–Radushkevitch (D–R) isotherm models in the studied concentration range at 25 °C;
- The obtained results from the equilibrium studies of the adsorption of thorium ions on NZ, PZ, and SZ demonstrated that these adsorbent materials were capable of removing thorium ions from aqueous solutions;
- The modification of natural zeolite with sulfate anions did not show a significant improvement of the adsorption capacity for thorium ions. However, the adsorption capacity was substantially enhanced by modifying it with phosphate anions, as the maximum adsorption capacity of thorium was found to be 17.27 mg-Th/ g- for phosphate-modified zeolite;
- The present research has clearly shown the value of phosphate-modified zeolite as an appropriate adsorbent material, which could potentially be used for the efficient removal of thorium from contaminated aqueous solutions.

## Figures and Tables

**Table 1:** The textural properties of adsorbent materials.

Adsorbent Materials	BET Surface Area (m^2^/g)	Average Pore Size (nm)	Total Pore Volume (cm^3^/g)
Natural zeolite (NZ)	28.84 ± 0.49	17.47	0.114
Phosphate-modified zeolite (PZ)	25.04 ± 0.58	19.11	0.116
Sulphate-modified zeolite (SZ)	20.62 ± 0.61	19.64	0.101

**Table 2:** The functional groups present in the natural zeolite.

No.	Bonding Type	Functional Group	Wavenumber (cm^−1^)
1	Stretching Vibrations	−OH	3454
2	Harmonic Vibrations	H-O-H	1628.5
3	External vibrations	(Si,Al)O_4_	1203.43
4	Stretching Vibrations	T-O (T=Si or Al)	1017.1

**Table 3:** The corresponding bands of the FTIR spectra for NZ, PZ, and SZ.

Samples	Corresponding Bands (cm^−1^)							
NZ	673.25	730.4	794.5	1017.1	1628.5	1739.7	3454	3602.4
PZ	675	728	794.8	1025.6	1628.5	1739.5	3431	3622.4
SZ	673.4	729	794	1020	1628	1739	3416	3601

**Table 4:** EDX analysis showing the elemental composition for NZ, PZ, and SZ.

Element	Natural Zeolite (NZ)	Phosphate-Modified Zeolite (PZ)	Sulphate-Modified Zeolite (SZ)
Weight%			
O	51.2	34.3	38.8
Si	34.1	44	39.2
Al	7.4	6.5	6
P	-	0.9	-
S	-	-	0.6
Ca	3.3	5	7
Na	0.9	-	0.4
Mg	0.4	-	-
K	2.7	9.3	7.9
Si/Al ration	4.6	6.7	6.53

**Table 5:** Isotherm parameters and the correlation coefficient values (R^2^) for the adsorption of thorium onto NZ, PZ, and SZ.

Adsorption Material	Langmuir Isotherm				Freundlich Isotherm			(D–R) Isotherm		
	Qo (mg/g)	K_L_ (L/mg)	R^2^	R_L_	n	K_f_ (mg/g)	R^2^	q_max_ (mg/g)	K (mol^2^/kJ^2^)	R^2^
NZ	10.21(12.7) ^a^	0.32	0.99	0.05–0.005	10.66	7.21	0.68	10.08	5.17 × 10^−9^	0.78
PZ	17.27 (17.4) ^a^	0.09	0.99	0.16–0.016	4.56	5.21	0.91	12.05	1.15 × 10^−7^	0.69
SZ	13.83 (13.8) ^a^	0.06	0.98	0.24–0.026	5.64	4.72	0.94	9.93	4.36 × 10^−8^	0.66

^a^ Values in parentheses refer to the experimental values.

**Table 6:** Comparison of the maximum adsorption capacity of thorium onto PZ and SZ with various adsorbents.

Adsorbent	Uptake Capacity (mg/g)	pH	Temperature (°C)	Adsorbent Mass (g/L)	Contact Time (min)	Reference
Synthetic zeolite	2.82	3.6	25	0.5	2880	[76]
1-(2-pyridylazo)-2-naphthol (PAN)/zeolite	9.28	4	25	3	45	[39]
Monomodified b-cyclodextrin polyrotaxane	12.92	4	25	1	35	[77]
Activated bentonite	14.3	2.5	45	0.3	1440	[22]
Merrifield polymer-DMDBMA	15.74	-	25	20	45	[78]
Zeolite	0.24	3	-	-	120	[23]
Mesoporous Al_2_O_3_	11.72	1	55	5	60	[79]
Natural zeolite (NZ)	10.21	3	25	15	1440	Present work
Phosphate-modified zeolite (PZ)	17.27					
Sulfate-modified zeolite (SZ)	13.83					

## Data Availability

Data is contained within the article.
